# CRISPR-mediated genome editing in non-conventional yeasts for biotechnological applications

**DOI:** 10.1186/s12934-019-1112-2

**Published:** 2019-04-02

**Authors:** Peng Cai, Jiaoqi Gao, Yongjin Zhou

**Affiliations:** 1 Division of Biotechnology, Dalian Institute of Chemical Physics, Chinese Academy of Sciences, 457 Zhongshan Road, Dalian, 116023 People’s Republic of China; 2 School of Life Science and Biotechnology, Dalian University of Technology, Dalian, 116023 People’s Republic of China

**Keywords:** CRISPR–Cas9, Non-conventional yeasts, Genome editing, Guide RNA, Homologous recombination, Non-homologous end joining

## Abstract

Non-conventional yeasts are playing important roles as cell factories for bioproduction of biofuels, food additives and proteins with outstanding natural characteristics. However, the precise genome editing is challenging in non-conventional yeasts due to lack of efficient genetic tools. In the past few years, CRISPR-based genome editing worked as a revolutionary tool for genetic engineering and showed great advantages in cellular metabolic engineering. Here, we review the current advances and barriers of CRISPR–Cas9 for genome editing in non-conventional yeasts and propose the possible solutions in enhancing its efficiency for precise genetic engineering.

## Background

Yeasts are extensively used for industrial bioprocesses and fundamental research with a long history. Owing to its distinguished tolerance of harsh cultivating conditions and convenient genetic manipulation, *Saccharomyces cerevisiae* becomes the most outstanding cell factory for manufacturing of vast chemicals, biofuels and natural products [1–3]. However, a number of non-conventional yeasts with different evolutionary distance to *S. cerevisiae* have increasingly attracted great attention for production of fine chemicals, oils and recombinant proteins [4, 5]. The specific natural characteristics of non-conventional yeasts, such as *Scheffersomyces* (*Pichia*) *stipitis*, *Komagataella phaffii* (*Pichia pastoris*), *Ogataea (Hansenula) polymorpha*, *Kluyveromyces lactis*, *Yarrowia lipolytica*, *Kluyveromyces marxianus*, *Ogataea thermomethanolica*, can bring great advantage for specific bioproduction processes. Compared to *S. cerevisiae*, *S. stipits* and *O. polymorpha* have complete xylose metabolic pathways, so that they are widely used for ethanol fermentation from biomass hydrolysates containing xylose and glucose [6, 7]. The methylotrophic yeasts, e.g. *K. phaffii*, *O. polymorpha* and *O. thermomethanolica,* are typically used for heterologous protein production, due to their high-efficient heterogeneous protein secretion and glycosylation [8–10]. *Kluyveromyces lactis* is widely used in food and feed industries because of its ability to metabolize lactose and high protein secretion [11]. The oleaginous yeast *Y. lipolytica* has the high ability to transform the carbon sources into cellular lipids [12]. The thermo-tolerance of *K. marxianus*, *O. polymorpha* and *O. thermomethanolica*, facilitate the process efficiency at higher temperatures such as simultaneous saccharification and fermentation, thus saving the cooling water and process time [13].

**Fig. 1:**
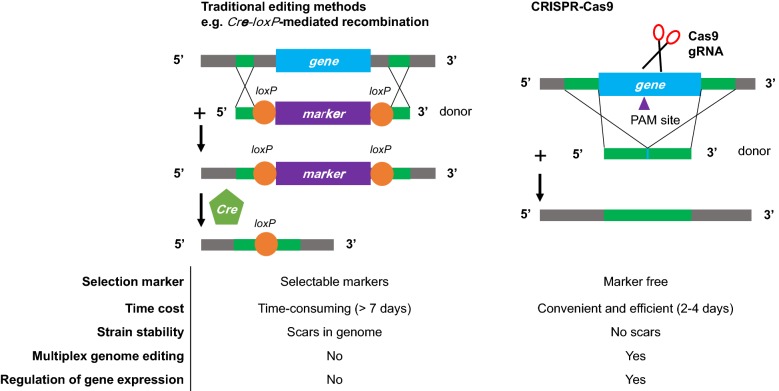
Comparing the conventional genome engineering with CRISPR-mediated genome editing. Conventional genome editing methods heavily rely on the use of selection markers for validation and maintenance of the integrated sequences. Furthermore, conventional techniques need multiple rounds of selection and screening to create and identify positive clones, which is time consuming, leave scars in the genome and reduce the genome stability. CRISPR-mediated genome editing system involve genome cutting and repair, which avoid selection marker integration and recycling. In addition, CRISPR–Cas9 system has the power of multiplex genome editing by cell native repair system. Besides, dCas9 system can be used to regulate the gene expression in metabolic engineering

In spite of so many excellent properties, it is still challenging in engineering these non-conventional yeasts due to serious lack of genetic editing tools in compared with the modeling yeast *S. cerevisiae* with numerous advanced genetic tools and biological devices [14]. The efficient genetic editing tools and methods are essential for rapid engineering cellular metabolism and robustness toward efficient synthesis of product of interest [15]. A crucial step in genome editing is the introduction of double-stranded breaks (DSBs) at the target loci. Afterwards, the DSBs can be repaired in two major patterns: non-homologous end joining (NHEJ) or homologous recombination (HR). In *S. cerevisiae*, HR plays a dominant role in DSBs repairing process, and 50 bp short homology arms is sufficient to bring nearly 100% target repair [16]. However, NHEJ is the dominant repairing mechanism in most other yeasts [15], which seriously hampers the precise rewiring the metabolic pathways in these non-conventional yeasts. Though some conventional genetic tools, such as *Cre*-*loxP* (Fig. 1) and split-marker technique, have been developed to improve the efficiency of precise genome editing, multiple round of marker selection and recycling are time consuming and some scars would be left in genome, which will bring genetic instability [17, 18]. In the past decades, several novel genetic manipulation tools have been developed for the precise genome editing. For example, the zinc-finger nucleases (ZFNs) [19] and transcription activator-like effector nucleases (TALENS) [20] were designed to cleave the specific DNA sequences with high accuracy, which however involve time-consuming and laborious construction of specific DNA binding proteins and thus are not suitable for simultaneous multiple target editing. Recently, Clustered Regularly Interspaced Short Palindromic Repeats and CRISPR-associated protein 9 (CRISPR–Cas9) system revolutionized the genome editing with the high efficiency, veracity and convenience [21–23]. Extensive applications of CRISPR–Cas9 system in *S. cerevisiae* have been reported and reviewed elsewhere [24, 25]. We here review the current advances on genome editing using CRISPR–Cas9 system in several non-conventional yeast species (Table 1). Furthermore, we discuss some strategies to improve the efficiency of CRISPR–Cas9 based genome editing and its feasible application in construction of non-conventional yeast cell factories.

**Table 1:** Genome editing applications of CRISPR–Cas9 in non-conventional yeasts

Yeasts	Cas9 promoter	sgRNA promoter	Host strains	Target locus	NHEJ efficiency (positive colonies/total colonies)	HR efficiency (positive colonies/total colonies), donor length	References
*S. stipiti*	*ENO1*	*SNR52*	UC7	*ADE2*	83%, (5/6)		[33]
				*TRP1*	100%,		
			UC7 *ku70*Δ*ku80*Δ	*ADE2*		71%, (5/7), 500 bp HA	[35]
				*TRP1*		64%, (14/22), 50 bp HA	
			UC7	*KU70*	100%, (4/4)		
				*KU80*	83%, (5/6)		
*K. phaffii*	*HTA1*	*HTB1*	CBS7435	*GUT1*	94%, (79/84)		[39]
				*AOX1*	95%		
				*GUT1*+*AOX1*	69%		
				*MPP1*	95%		
				*TRM1*	94%		
				*MXR1*	43%		
				*OCH1*	51%		
			CBS7435 *ku70*Δ	*GUT1*		91%, 1000 bp HA	[41]
*O. polymorpha*	*TDH3*	*tRAN* ^*CUG*^	BY4330	*ADE12*	45%, (48/106)	47%, (186/394), 60 bp HA	[42]
	*AaTEF1*	*ScTDH3*	CBS4732	*ADE2*	9%		[43]
	*ScTEF1*	*ScSNR52*		*LEU2*	58%		[45]
				*URA3*	65%		
				*ADE2*		62%, 1000 bp HA	
				*URA3*+*HIS3*+*LEU2*		24%, 1000 bp HA	
*O. thermomethanolica*	*AOX*	*AOX*	TBRC656	*HAC1*	63%, (15/24)		[47]
				*MAL1*	97%, (126/130)		
				*MAL2*	93%, (37/40)		
*K. lactis*	*FBA1*	*SNR52*	ATCC8585 *Gal80*Δ- *Ku80*Δ	*DIT*1+*ADH*1+*NDT80*		2%, 500 bp HA	[49]
*K. marxianus*	*ScPDC1*	*ScSNR52*	NBR1777 *Nej1*Δ	*URA3*		84%, (18/19), 120 bp HA	[52]
			NBR1777 *Dnl4*Δ			100%, (20/20), 120 bp HA	
			NBR1777 *Nej1*Δ	*Sed1*		84%, (6/7), 50 bp HA	
			NBR1777 *Dnl4*Δ			92%, (46/50), 50 bp HA	
	*TEF1*	*RPR1-tRNA* ^*Gly*^	CBS6556	*XYL2*		66%, (68/90)	[53]
*Y. lipolytica*	*UAS1B8-TEF1*	*SCR1’-* *tRNA* ^*Gly*^	PO1f (ATCC MYA-2613)	*KU70* *PEX10* *MFE1*	100% 54%, (16/30) 90%, (27/30)		[55]
			PO1f *Ku70*Δ	*PEX10* *MFE1*		86%, (30/35), 1000 bp HA 100%, (7/7),	
			PO1f	*MFE1* *AXP* *XPR2* *XDH* *XYR*		$$ \left. \begin{aligned} 69\% , \hfill \\ 62\% , \hfill \\ 48\% , \hfill \\ 53\% , \hfill \\ 52\% \hfill \\ \end{aligned} \right\}1000 \; {\text{bp}}\;{\text{HA}} + {\text{GFP}} $$	[59]
	*TEFin*	*TEFin*	PO1f	*TRP1*	13%, (10/76)		[55]
			PO1f *Ku70*Δ PO1f *Ku70*Δ*Ku80*Δ			$$ \left. \begin{aligned} 1 1\% , \, \left( { 1 1/ 9 8} \right) \hfill \\ 100\% , \, \left( { 1 9/ 1 9} \right) \hfill \\ 100\% , \, \left( { 1 5/ 1 5} \right) \hfill \\ \end{aligned} \right\} 500{\text{ bp HA}} $$	[58]
			PO1f	*PEX10*		$$ \left. \begin{aligned} 6 1\% , \, \left( { 1 7/ 2 8} \right) \hfill \\ 3 3\% , \, \left( { 4/ 1 2} \right) \hfill \\ \end{aligned} \right\} 500{\text{ bp HA}}\; {\text{in plasmid}} $$	
				*TRP1+PEX10*		37%, (15/40)	
				*TRP1+PEX10+GUT2*		20%, (7/35)	

## Mechanism of the CRISPR–Cas9 system

**Fig. 2:**
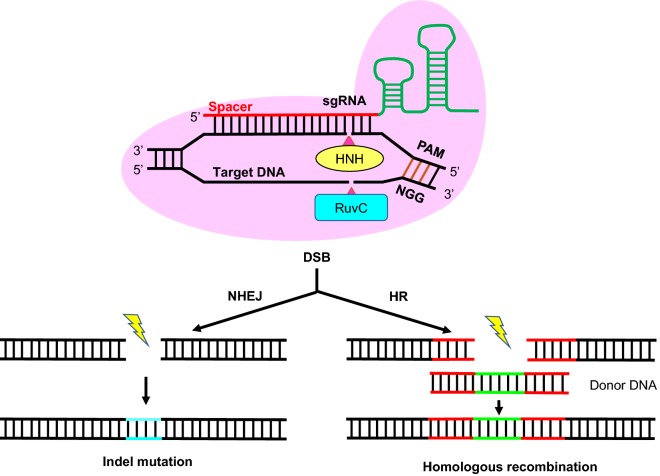
Overview of the CRISPR–Cas9-mediated genome editing system. The Cas9 and sgRNA form a complex in vivo and then bind on the target DNA sequence upstream of PAM sequence. The Cas9 nuclease domain HNH then cleaves the target DNA sequence complementary to the 20 bp guide sequence, while RuvC domain cuts another DNA strand, forming a DSB. DSB must be repaired via either NHEJ or HR immediately to avoid cell death

CRISPR–Cas system was first discovered to provide the immunological weapon for bacteria and archaea against invading bacteriophages (viruses) and mobile genetic elements [26, 27]. CRISPR–Cas systems are categorized into two distinct classes (six types) based on effector module organizations. In particular, the type II CRISPR system from *Streptococcus pyogenes* has been extensively studied and well characterized [28, 29], and it is also the most commonly used in yeast genetic engineering. The Cas9 protein is a RNA-mediated endonuclease, cleaving the double DNA strands with two active parts—HNH domain and RuvC domain (Fig. 2). Since Cas9 was identified from bacterium, a nucleus localization sequence (NLS) needs to be fused to Cas9 to allow targeting the eukaryotic nucleus genomes [15]. Another necessary component is single guide RNA (sgRNA) that guides Cas9 to target sites. The canonical sgRNA consists of a CRISPR targeting RNA (crRNA) and a trans-activating crRNA (tracrRNA). The first 20 base pairs complementary sequence at 5′ end of crRNA is indispensable for Cas9 endonuclease function, and three nucleotides protospacer adjacent motif (PAM) NGG must be found immediately at 3′ end of the desired locus in genome [30]. The sgRNA has a specific secondary structure to recruit Cas9 to form a functional complex. Following the guide of sgRNA, Cas9 target the genome specific sequence with PAM and cleave the both strands of DNA [31]. Once the introduction of DSBs, the DNA repairing process should proceed to prevent cell death. Normally, NHEJ repair is considered to generate gene disruption by insertion or deletion (indel) mutation and HR repair allows for the replacement or insertion of desired sequences with the existence of donor DNA (Fig. 2).

## CRISPR–Cas9 mediated precise genome editing in non-conventional yeasts

Drawing on the successful experiences of *S. cerevisiae*, CRISPR–Cas9 system has been already applied in several non-conventional yeasts. Though some system is waiting for further optimization, this system has showed great potential in genome editing in non-conventional yeasts.

### *Scheffersomyces stipitis*

*Scheffersomyces stipitis* is one of the most notable microorganisms for biomass refinery due to its excellent native capacity for catabolizing xylose. Furthermore, it shows great potential for producing shikimate pathway derived molecules [32]. Establishing the CRISPR–Cas9 system encounters the challenge for lack of the stable and useful plasmid to express the CRISPR components. Recently, a 500 bp minimal fragment of centromere (CEN) was identified to significantly stabilize the autonomously replicating sequences (ARS)-containing vector and enable exogenous gene expression [33]. Then a codon-optimized version of Cas9 gene for *S. stipitis* was fused with nucleus targeting signal NLS at both ends and was expressed under the control of the constitutive *ENO1* promoter and the *TEF1* terminator. A native RNA polymerase III *SNR52* promoter was used for functional expression of guide RNA (gRNA). This established CRISPR–Cas9 system enabled up to 80% of gene disruption with indel mutations when targeting to *ade2* and *trp1* genes [33].

A simple indel mutation based on NHEJ repair mechanism is not preferred in precise genome editing at the DSB site duo to its non-predictability in precise pathway engineering. Alternatively, HR-mediated genome modification will facilitate the precise genome modification. In yeasts, the complex of *Ku70* and *Ku80* could bind to the DSB site to facilitate the NHEJ repair process and eliminating these two genes can repress the NHEJ and enhance the HR [34]. Transformation of different lengths of homologous arms (HAs) into *ku70Δ*/*ku80*Δ strain together with the Cas9 plasmid carrying sgRNA of *trp1*, resulting in a high HR efficiency between 73 and 83% (Table 1) [35]. Despite the extremely decreased numbers of transformants, the HR editing efficiency was improved about fourfold at *trp1* and *ade2* sites in *ku70*Δ/*ku80*Δ background in compared to the parental strain. Other than repressing NHEJ, enhancing HR efficiency by expressing HR associate Rad protein would be another approach. However, introducing the codon-optimized *rad51* and *rad52* from *S. cerevisiae* into in *Ku* deleted *S. stipitis* had no obvious improvement in enhancing HR [35].

### *Komagataella phaffii*

*Komagataella phaffii* is widely used as a host for the production of recombinant proteins [36] and recently is attracting great attention as a cell factory for production of chemicals [37, 38]. However, the lack of genome editing tool and the poor HR efficiency make this methylotrophic yeast hard to be engineered. Different with *S. cerevisiae*, NHEJ plays the preponderant role in *K. phaffii*. Recently, CRISPR–Cas9 system was established and optimized in *K. phaffii* by evaluating diverse codon-optimized Cas9 genes, sgRNA and promoters for expression of the Cas9 and sgRNA [39]. Expression of a human optimized Cas9 and HH/HDV ribozyme flanked sgRNA under the control of native bidirectional *HTX1* promoter, resulted in an up to 90% NHEJ efficiency in *GUT1* disruption. Application of this optimized system for targeting other five different genes (*AOX1*, *MXR1*, *TRM1*, *MPP1* and *OCH1*) resulted high disruption efficiencies of 50–100%. Further simultaneous disruption of *GUT1* and *AOX1* by transforming the Cas9 plasmid with two sgRNAs, led to an up to 69% mutation efficiency [39].

Though DSBs can drastically increase specific integration [40], introducing the donor cassette with 1 kb homologous arms into the Cas9 cutting loci only provided a very poor integration efficiency of 2.4% [39]. To overcome the barrier of low frequency of HR, disruption of the NHEJ repairing gene *ku70* enabled a nearly 100% HR disruption efficiency with markerless donor cassettes. Interesting, adding an ARS to the donor DNA, significantly enhanced the HR integration efficiency [41], which might be attributed to the improvement of the stability of donor DNA in vivo.

### *Ogataea polymorpha*

The methylotrophic yeast *O. polymorpha* is not only recognized as a promising cell factory for producing heterologous protein, but also a model organism in studying the methanol metabolism. Furthermore, *O. polymorpha* has great potential in industrial application field due to its characteristics of thermostability and fast growth [6]. To establish the CRISPR–Cas9 editing system, a human codon-optimized Cas9 gene was first cloned into a plasmid under the control of *S. cerevisiae TEF1* promoter (*ScTEF1p*) and its terminator (*ScTEF1t*) [42]. The targeting sgRNAs (*ADE12*, *ADE8*, and *PHO85*) were expressed under the small noncoding RNA promoter (*OpSNR6*). Unfortunately, this CRISPR–Cas9 system got less than 1% gene disruption efficiency. Further adding a Hyg resistance marker (hphNT1) to a 60 bp homologous arms improved the disruption efficiency up to 47%, which suggested sgRNA was not well expressed for guiding Cas9 toward targeting loci. Thus, a modified system with tRNA^CUG^-sgRNA fusion cassette was used to improve the sgRNA function, which significantly enhanced the indel mutations to 17–71% when disrupting *OpPHO*1, *OpPHO*11 and *OpPHO*84 [42]. A broad-host-rage CRISPR–Cas9 system was constructed by using high-fidelity *Sp*Cas9^D147Y P411T^ for gene disruption of *ADE2* in four non-conventional yeasts *K. lactis*, *K. marxianus*, *O. polymorpha* and *O. parapolymorpha*, which however obtained 9% of mutations in *O. polymorpha* when targeting *ADE2* loci [43]. The low gene disruption efficiency might be attributed to the insufficient expression of Cas9 and sgRNA under the heterologous *AaTEF1* promoter from *Arxula adeninivorans* and *ScTDH3* promoter from *S. cerevisiae*.

The more than 50 copies of long homologous sequences in the rDNA locus, provide sufficient integration sites for genome expression of high copy of heterologous genes [44]. Taking advantage of this characteristics, a CRISPR–Cas9-assisted multiplex genome editing (CMGE) approach was developed for polygenic knockout and multiplex gene integration at multi sites with multi copies in *O. polymorpha* [45]. In this study, Cas9 and sgRNA expression cassettes were inserted into the genome of *O. polymorpha* due to the lack of available and stable expression vectors. This system enabled 58% and 65% disruption efficiencies of *OpLEU2* and *OpURA3* respectively, by using a repairing cassette of 1.5 kb homologous arms, and a 24% mutation when simultaneously knocking out of *URA3*, *HIS3* and *LEU2* genes. CMGE system also achieved a precise point mutation of *URA3* (G73T) with the editing efficiency of 31%. At last, CMGE-MC enabled the integration of more than 10 copies of GFP mutation (*gfpmut3a*) into the rDNA sites in *O. polymorpha*, and the multi-copy of integration can be stably maintained after cultivating for 55 generations [45].

### *Ogataea thermomethanolica*

Like *O. polymorpha*, *O. thermomethanolica* is also a thermotolerant methylotrophic yeast and widely used to produce heterologous proteins [46]. Since no RNA polymerase III promoter has been found in *O. thermomethanolica*, the native inducible AOX promoter was selected to express Cas9 protein and sgRNA in an integrative plasmid. This system was applied for editing the three sugar metabolism relating genes *OtMAL1* (maltase), *OtMAL2* (maltose permease) and *OtHAC1* (UPR regulator) with efficiencies of 97% and 93% and 63%, respectively [47]. Another episomal CRISPR–Cas9 system in *O. thermomethanolica* was developed in order to perform various rounds of genome editing by using an ARS element from *K. lactis*, resulting in the mutation efficiency of 92% [47]. It is regretful that this genome editing tool is based on NHEJ repair and there has been no report on HR repair via CRISPR–Cas9 system in *O. thermomethanolica* so far.

### *Kluyveromyces lactis*

*Kluyveromyces lactis* is a widely used host in fundamental research and industrial production of various chemicals, pharmaceuticals and enzymes [48]. The scarcity of selection markers makes it time-consuming in marker recycling during traditional genome editing [48]. Thus, CRISPR–Cas9 editing system was the first established in this non-conventional yeast [49]. *Cas9* gene was integrated at *GAL80* site by using the medium-strength promoter *FBA1p* and *ku80* was deleted to minimize the NHEJ effect. The typical *SNR52* pol III promoter and *SUP4* terminator were used to express gRNA and an episomal expression system was constructed by inserting another stabilizing element pKD1 to a *S. cerevisiae* 2 μ plasmid. This genome editing system successfully integrated donor DNA with 1 kb flanking arms to *DIT1*, *ADH1* and *NDT80* locus, though with a low triple integration efficiency of only 2% [49]. As mentioned above, a broad-host-rage CRISPR–Cas9 system worked well in *K. lactis*, where a 962 bp repair donor enabled a 31% HR based disruption of *ADE2* [43].

### *Kluyveromyces marxianus*

*Kluyveromyces marxianus*, a non-conventional thermotolerant yeast, is known as its fast growth and Crabtree-negative property, is considered as an ideal host for production of diverse chemicals and bioactivities [50]. Since NHEJ plays the main role in *K. marxianus*, relatively long homologous arms are needed for HR editing [51]. To repress the NHEJ effect, a stop codon was introduced to the in NHEJ core genes of cell-type specific regulator (*Nej1*) and DNA ligase 4 (*Dnl4*) by changing C to T at 16 to 19 bp upstream of PAM, which enabled a 100% correct HR based genome editing at *URA3* site by using a zeocin selection marker harboring 1 kb homologous arms, representing fourfold improvement compared with wild-type strain [52]. To test the markerless integration with CRISPR–Cas9, 50 bp homology arms donor result in a nearly 100% deletion/null mutation efficiency at the *Sed1* target locus in this NHEJ repressed strain, which was also significantly higher than that of wild type hosts (38%) [52]. Similarly, a broad-host-rage CRISPR–Cas9 system was applied for *K. marxianus* haploid and diploid strains, which resulted a more than 80% disruption for *ADE2* disruption with a 24% HR based repair [43]. The low HR efficiency again suggested the NHEJ played the main role in DSB repair.

This CRISPR–Cas9 system was also applied to characterize functional genes in biosynthesis pathway of ethyl acetate and ethanol in *K. marxianus* [53]. Three types of hybrid pol III promoter, including SNR52-tRNA^Gly^, SCR1-tRNA^Gly^ and RPR1-tRNA^Gly^, were used to ensure functional expression of various sgRNAs, and RPR1-tRNA^Gly^ promoter showed the highest editing rate of 66%. Screening of the disruption genes of alcohol dehydrogenase (*ADH*) and alcohol-*O*-acetyltransferase (*ATF*) genes, revealed that *ADH7* played the main role as an alternative pathway for ethyl acetate biosynthesis. This study showed a good example that CRISPR–Cas9 system can help to rapidly construct gene disruption sets for functional characterization of hindered pathways and genes that were involved in synthesis of some valuable chemicals.

### *Yarrowia lipolytica*

*Yarrowia lipolytica*, a generally recognized as safe (GRAS) microbe, is the most studied oleaginous yeast and has been worked as an industrial host for production of lipase, fragrances, citric acid, omega-3 fatty acids and carotenoids for decades [54]. Like the other non-conventional species, metabolic engineering in this yeast is hindered by low HR efficiency and insufficient genetic tools. Recently, CRISPR–Cas9 based genome editing demonstrated the potential in rapid genome modifications in this yeast.

Schwartz et al. [55] developed a pCRISPRyl plasmid to carry the Cas9 gene and the sgRNA together. The *Y. lipolytica* codon optimized Cas9 with a C-terminal SV40 NLS fusion was expressed under the constitutive and strong hybrid promoter UAS1B8-TEF(136) [56]. To express the sgRNA, three synthetic Pol III promoters, RPR1-tRNAGly, SCR1-tRNAGly, and SNR52-tRNAGly were designed and tested. This system enabled a 54% deletion efficiency of *PEX10* gene via NHEJ after 2 days cultivation and a more than 92% deletion efficiency by using SCR1-tRNAGly promoter for sgRNA expression when the culture time was extended to 4 days. This system also enabled > 90% efficiency for deletion of *KU70* and *MFE1*. Enhancing the HR efficiency by disrupting *KU70* enabled a 100% correct integration at *MFE1* locus by using hygromycin as a selection marker [55]. This gene knockout system had been successfully used as a fast and effective method to determine functional candidate genes in xylose metabolic pathway. Combined with gene overexpression, the results showed that *XDH* and *XKS* was essential for xylitol metabolism [57]. However, the selection marker should bring another around work of marker removing. An alternative CRISPR–Cas9 genome editing system, using constitutive RNA pol II promoter TEF1 for expression of Cas9 and the gRNA, enabled double and triple gene deletions with 37% and 19% efficiencies in a *ku70*/*ku80* double deleted strain [58]. The multiple gene deletion should be helpful in metabolic engineering, but the efficiency is waiting for further improvement.

Other than gene knockout, genome integration is very important for introducing heterologous genes or pathways during cell factory construction. To identify suitable integrations sites without influencing cell viability, humanized Renilla GFP (*hrGFP*) cassettes with 1 kb homology arms were targeted to 17 different loci via CRISPR–Cas9 associated HR with about 60% targeting efficiency [59]. This multi-gene integration system should serve as a valuable scarless genome integration platform, rather than traditional *Cre*-*loxP* recombination system that leaves a scar in the genome after marker recycling (Fig. 1).

## Construction of cell factories with CRISPR–Cas9 in non-conventional yeasts

Distinct metabolic advantages, such as high metabolic flux in TCA cycle, strong amino acid synthesis ability and powerful protein secretion, make the non-conventional yeasts more outstanding hosts for some specific bioprocesses [60, 61]. With the ever-increasing wealth of omics information, CRISPR–Cas9 systems can help the construction of cell factories for improved production of chemicals by speeding up the genetic writing.

**Fig. 3:**
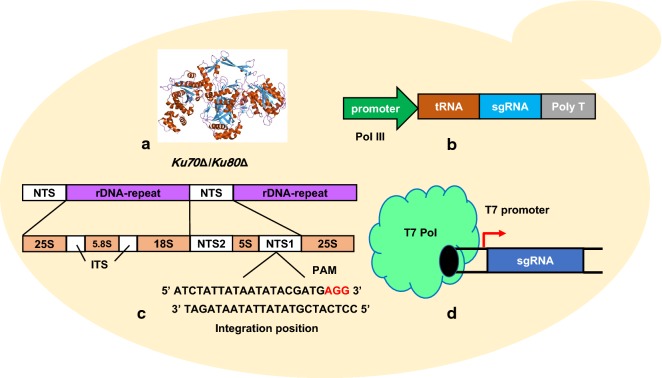
Optimizing strategies of CRISPR–Cas9 system in genome editing of non-conventional yeasts. **a** The Ku70/80 heterodimer is regulatory DNA-binding subunits of DNA-dependent protein kinase (DNA-PK), which is the main component of the NHEJ system in eukaryotes. Knockout the *Ku70* and *Ku80* genes can repress the NHEJ system. **b** Synthetic promoters are generated by placing the pol III promoter sequences immediately upstream of the tRNA. **c** rDNA tandem array can serve as target loci for multi-copy integrations due to its high copy numbers of head-to-tail repeats. **d** The T7 polymerase system (T7 polymerase and T7 promoter) can express the sgRNA and enable CRISPR-based genome editing in yeasts

With the aid of CRISPR–Cas9 based multiplex genome editing, the resveratrol biosynthetic pathway (three genes) was integrated into rDNA repeats cluster of *O. polymorpha* (Fig. 3c). This multi-copy pathway integration enabled a 21-fold higher resveratrol production (97 mg/L) compared to the single copy pathway [45]. Multiple integration of *cadA* gene from *E. coli* and the human serum albumin gene *HAS*, also enabled higher product synthesis. However, this single site multiple integration has some challenges in construction of long biosynthetic pathways with more than three genes. Thus, Schwartz et al. screened five integration sites that were suitable for construction of long pathways in *Y. lipolytica*. The lycopene biosynthetic genes, *Y. lipolytica* codon optimized *crtB* and *crtI* from *Pantoea ananatis*, native *HMG1* and *GGS1*, and *crtE* from *P. ananatis*, were integrated into the five identified sites separately, which enabled a 1.34 mg lycopene/g DCW, representing 8.6-fold increase in compared to the wild-type strain [59]. In cellular pathway engineering, gene tuning other than knockout might be beneficial for overall biosynthesis efficiency and cellular robustness. Thus, multiple CRISPR interference (CRISPRi) system was developed and applied for redirecting carbon flux of central metabolic pathways toward ethyl acetate production in *K. marxianus*. Fine regulated expression of genes of TCA cycle, electron transport chain, ethanol biosynthesis and acetyl-CoA supply, increased ethyl acetate titer by 3.8-fold [62]. Alternatively, CRISPR–dCas9 activation (CRISPRa) system was also developed in *Y. lipolytica*, which successfully activated native β-glucosidase expression and enabled *Y. lipolytica* growing on cellobiose as single carbon source [63].

In spite of demonstrating the promising potential as cell factories, the genetic engineering tools in these non-conventional yeasts is serious limiting for rapid and precise metabolic engineering. Thus, developing more efficient CRISPR based genetic engineering tool is greatly urgent.

## Feasible ways to optimize CRISPR–Cas9 system in non-conventional yeasts

As mentioned above, CRISPR–Cas9 system has already been reported in these non-conventional yeasts, there are still many obstacles to overcome, such as low HR efficiency, lack of native available RNA promoters, limitation of the NGG PAM motif, off-target effect and so on. Some recent studies provided the possibility of improving the efficiency of genome editing with CRISPR–Cas9.

### Increasing homologous recombination efficiency

Right now, CRISPR–Cas9 system is much less efficient in non-conventional yeasts compared to mammal and *S. cerevisiae,* which might be attributed to relative low HR repair efficiency. Once DSBs occurs, most of the non-conventional yeasts prefer NHEJ pathway over HR even with exogenous donors, which retards the precise genome editing. To overcome this barrier, NHEJ can be repressed by deleting its core component genes such as *ku70* and *ku80* [34] (Fig. 3a). Alternatively, it is easy to come out the idea that enhancing HR by overexpressing its component genes such as *Rad51/Rad52* complex. However, overexpressing the codon-optimized *ScRad51/Rad52* in *S. stipitis* had no obvious improvement in HR efficiency [35]. Presumably, the expression strengths of Rad protein should be fine-tuned at a suitable level. In addition, Charpentier et al. identified a minimal HE domain (N-terminal fragment of CtIP from aa 1 to 296) as HR enhancer. Fusion this HE domain to Cas9 (Cas9-HE) increased the HR efficiency by over twofold [64]. These results showed that the HR dependent repair can be enhanced by expression of the HR associated proteins or inhibition of NHEJ pathway.

### Improving the gRNA expression

In some microorganisms, guide RNA expression is insufficient and would limits the CRISPR–Cas9 targeting efficiency due to lack of suitable promoters. For gRNA expression, the promoter should be appropriate strong and will not introduce too much redundant nucleic acid sequence that will affect the binding efficiency of gRNA. The RNA pol III promoters are such good candidates for gRNA expression, however, it is failed to find suitable RNA pol III promoters in some hosts. Synthetic or hybrid promoters provide a feasible substitute for gRNA expression when it was absent of suitable natural RNA pol III promoters [55] (Fig. 3b). Recently, a T7-based artificial promoter was successfully developed for gRNA expression in yeast. In this system, a modified version of the T7 polymerase mutant (P266L) was fused with an SV40 NLS to ensure a functional T7 promoter for sgRNA expression, which showed a broad application in *S. cerevisiae*, *K. lactis* and *Y. lipolytica* with > 60% genome editing [65] (Fig. 3d). The reconstructed bacteria T7 system provide a feasible tool for sgRNA expression when the host has no suitable promoters.

### Expanding the recognition motif PAM

Targeting Cas9 protein to the specific DNA site requires the recognition of a PAM sequence. The recognition of NGG PAM by canonical SpCas9 occurs on average only about one in every 16 randomly chosen genomic loci [66]. One potential strategy to enhance editing scope is to relax the PAM recognition specificity of Cas9. Kleinstiver et al. designed an unbiased genetic method to engineering Cas9 variants with broader PAM recognition specificities. The engineered KKH SaCas9 showed activities toward broad PAM sequences of NNNRRT [66]. Moreover, the same group also identified and characterized a SpCas9 variant with an improved recognition pattern, which demonstrated superior characteristic against off-target effect with non-canonical NAG and NGA PAMs [67]. In another report, Hu et al. used the phage-assisted continuous evolution (PACE) technology to accelerate the evolutionary process. Then the most powerful version of SpCas9 variant (xCas9 3.7) achieved a 9.4-fold improvement in DNA targeting scope by recognizing a broad range of PAM sequences including NG, GAA, and GAT [68]. Though these Cas9 variants were tested only in human cells, they still have the potential to be used in non-conventional yeasts.

### Decrease of the off-target effect

The greatest challenge for the application of CRISPR–Cas9 system in genome editing is off-target effect that can bring unwanted sequence cleavage. The potential off-target effect should be detected to increase the cutting efficiency at desired locus. With the wide application of CRISPR–Cas technology, several in silico tools have been developed to design sgRNAs [24]. For example, Zhang Lab (https://zlab.bio/guide-design-resources) has developed a series of advanced tools to guide design with several non-conventional genome references, including the widely used CHOPCHOP and CasOFFinder [69]. The free website tools were well summarized in former literatures [24, 70]. Taking advantage of these online tools may largely increase the successful rate of the CRISPR-mediate gene editing.

## Conclusion

In general, it prefers to integrate a multi-gene pathway into the host and execute dynamic regulation for biotechnological application. Precise and maker free genome editing with CRISPR-Cas9 has shown great potential in synthetic biology and industrial biotechnology. To date, simultaneous multiple (locus or copy number) genetic editing and even accurate single base substitution have already accomplished in the model microbes such as *S. cerevisiae*, but lags behind in other non-conventional yeasts. Genome engineering in these non-conventional yeasts still relies on conventional genetic engineering tools. For example, the linearized plasmid was used to construct the de novo production pathway of monacolin J and lovastatin in *K. phaffii* [71], which is time consuming and limit the landscape for large scale genome modification. To overcome the obstacles, various CRISPR–Cas9 systems have been established and optimized for genome engineering by enhancing the HR process and developing more credible gRNA promoters, etc. Though the efficiency is still much lower than *S. cerevisiae,* the strategies or ideas that were developed in *S. cerevisiae* could shed light for optimizing the CRISPR–Cas9 systems in non-conventional yeasts. We believe that the genome editing system will be further refined and facilitate the creation of the desired phenotypes in non-conventional yeasts for industrial bioprocess.
